# AI and its consequences for the written word

**DOI:** 10.3389/frai.2023.1326166

**Published:** 2024-01-04

**Authors:** Thomas Hellström

**Affiliations:** Department of Computing Science, Umeå University, Umeå, Sweden

**Keywords:** ChatGPT, societal impact, LLM, Large Language Models, AI, human writing, the written word

## Abstract

The latest developments of chatbots driven by Large Language Models (LLMs), more specifically ChatGPT, have shaken the foundations of how text is created, and may drastically reduce and change the need, ability, and valuation of human writing. Furthermore, our trust in the written word is likely to decrease, as an increasing proportion of all written text will be AI-generated – and potentially incorrect. In this essay, I discuss these implications and possible scenarios for us humans, and for AI itself.

## 1 Introduction

There is something special about words. The recently developed *Large Language Models* (LLMs), which are exceptionally proficient at writing and reading, are also claimed to be “a spark of Artificial General Intelligence …” (Bubeck et al., 2023), “self-conscious” (Scott et al., 2023), and even “an existential threat to humanity.”^1^ At the same time, or rather for several decades, we have had computers that add and multiply numbers several billion times faster than any human. Doing math is also very “General,” but these computers have never been seen as particularly intelligent, at least not compared to us humans. So there seems to be something special also about machines that can handle words.

This paper, which is a continuation of the opinion piece (Hellström, 2023), examines what the widespread usage of LLMs will mean for us humans as writers, and for our view of the written word. The LLM-driven chatbot *ChatGPT* was launched by the company OpenAI at the end of 2022. Since then, several competing products have been released, for example, *Bard* (Google), *Ernie* (Baidu), and *Claude* (Anthropic). However, ChatGPT rapidly reached over 100 million users (Hu, 2023) and currently has a market-leading position. For this reason, we will in the following most often refer to ChatGPT, even if the discussion is believed to be generally valid for all chatbots driven by LLMs.

The paper begins with a general analysis of the relation between our use of language and intelligence. Old and new ways of creating and communicating the written word are then analyzed. We then move on to the possible short- and long-term effects of using LLMs, with separate sections on the amount of human writing, the human skill to write, the valuation of this skill, and our faith in the written word. To support an analysis of where we are heading, the limitations of, and expected problems with, LLMs are then discussed. Bringing it all together, we then look into possible future scenarios. An apocalyptic scenario paints a future where not only human writing has declined and transformed in unfortunate ways, but also our ability to think is negatively affected by machines doing too much of our intellectual work. A less apocalyptic scenario describes how AI helps us humans to simplify and improve our writing. While other social consequences of the usage of LLMs also are expected to be huge, we do not discuss them further in this paper. For a review and discussion of these aspects see for example (Farina and Lavazza, 2023; Sætra, 2023).

## 2 The relation between use of language and intelligence

The Swedish poet Esaias Tegnér pointed out a connection between the use of words and thinking, with the famous expression “what is dimly said is dimly thought.” However, our current praise of the LLMs rather suggests that we invert this into something like “what is clearly said is clearly thought.” So, when ChatGPT writes something well formulated, we infer that it is therefore also intelligent. Evidently, this is not a logically valid inference, but the conclusion may, of course, be correct anyway. There are indeed several connections between advanced usage of language and intelligence. For one, it is unique for us humans, and it serves as an effective and efficient tool to express and communicate thoughts. Furthermore, a talking chatbot creates the feeling that “there's someone on the other side,” in a way that a computer that spits out thousands of numbers with lots of correct decimals rarely does. And then we have, of course, the *Turing Test* (Turing, 1950). Alan Turing, the founder of theoretical computer science and AI, suggested this test that has become something of a gold standard by which machine intelligence is assessed. The general idea is as follows:

- *B* is a human and *A* is a computer that can communicate using written natural language.
- A human tester *C* communicates with *A* and *B* in writing but cannot see which one is human or computer.
- *C* freely asks *A* and *B* questions through written notes.
- *A* and *B* response in writing.
- *C* should determine which one of *A* and *B* is a machine and which is a human.
- *A* is considered “thinking” or “intelligent” if *C* fails.

If *A* is really bad at answering, *C* is right 100% of the time, and if *A* is at the level of *B, C* is right 50% of the time. When a variant of the Turing test was conducted with *A* being a computer running GPT-3 (which is an early version of the LLM inside ChatGPT), *C* was right 52% of the time (Brown et al., 2020). Judging by this test, GPT-3 is very close to being intelligent at the level of a human. Without engaging in the discussion of whether this is true, we conclude that the ability to create and understand natural language is closely connected to our notion of intelligence – human as well as artificial.

As a side note, the written word has not always been so highly regarded. Socrates believed that spoken discourse was the only way to transmit true knowledge between people, and writing and reading were both ineffective and harmful (Plato, 2009). However, the reason we know Socrates' thoughts is that his student Plato wrote them down and it is fair to say that Socrates' view of the written word has not stood the test of time very well. Technical innovations, such as the printing press and computers, have enabled the spread of written ideas via both paper and the Internet in an extremely efficient way (although Socrates might have objected that no ideas were actually spread at all).

## 3 Different ways of creating and spreading the written word

**Figure 1:**
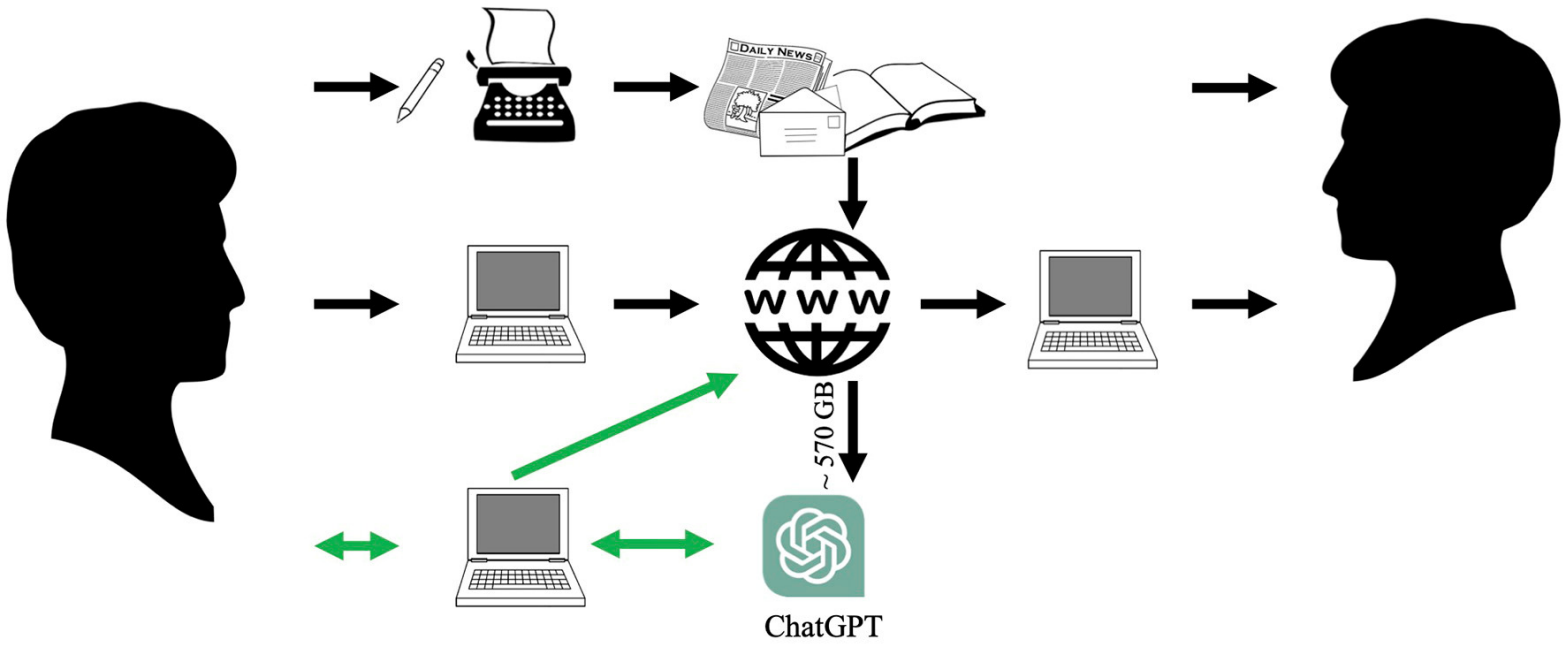
Different ways of generating and transmitting thoughts in writing between people, via paper and via the Internet. ChatGPT, which was trained on ~570 GB of existing text, is increasingly used as a tool to generate text to be communicated via the Internet.

The different ways humans historically have generated and transmitted written thoughts from a sender to one or several receivers are illustrated in Figure 1. At the top, we see how thoughts were written on, or transferred to, paper, which was then distributed and read by the receiver(s). Paper was then largely replaced by electronically stored text, input by the sender, and transmitted via the Internet to the receiver(s) (center).

At the bottom, the most recent development is depicted, wherein LLM-driven chatbots, like ChatGPT, serve as tools for text generation. ChatGPT was originally trained using about 570GB of filtered text data from the Internet (Brown et al., 2020), either directly generated by humans or computers, or scanned versions of text sources in paper form. Based on all the text it was exposed to during training, it generates responses to “prompts,” text-based instructions, provided by the sender. The sender may then choose to edit or otherwise use the response and upload the result to the Internet where it can be accessed by the receiver(s). With appropriate prompts, ChatGPT can generate many kinds of text, for example, social media posts, emails, blog articles, and overviews of research areas. By including text from other contexts in the prompt, ChatGPT can produce, for example, summaries, inferences, comparisons, sentiment analysis, and translations to other languages. ChatGPT can also generate answers to given questions, write computer programs, and pretty much anything that relates to text generation (for a more comprehensive overview of what ChatGPT can do, see, for example, Farina and Lavazza, 2023).

LLM-based tools may be developed for specific applications, such as fiction writing. Back in 2016, long before ChatGPT, the partially AI-written short story “The Day a Computer Writes a Novel” received high scores from a competition jury that praised both structure and action (Sato, 2016), and more recently several books written entirely with AI have been published. But above all, there are lots of AI-based writing aids^2^ that, for example, can write a story based on a brief description of the desired content, language style, and the intended readership. Furthermore, the new technology enables new forms of writing, such as *immersive* and *interactive storytelling*,^3^ in which readers can make choices that influence the direction and outcome of the story. This may create a sense of agency and immersion that traditional storytelling often lacks. AI can also help in the creation of personalized content by adapting the produced text to the reader's interests and needs (see footnote 3). This goes beyond the common use of “algorithms” to *select* content, by actively *creating* content that fits the reader.

Computers have, even before the recent AI developments, been used for “automated journalism”^4^ (Canavilhas, 2022), to write,^5^ for example, sports reports, traffic accident notices, weather reports, biographies, obituaries, and press releases. LLM-based systems have the potential to be more advanced by their ability to work with unstructured data sources, identify and compile background information, and draw parallels with past events.

## 4 How the usage of LLMs will affect writing

In this section, we discuss how the introduction of LLMs has affected, and will continue to affect, four aspects of human writing: the amount of human writing, the human ability to write, the valuation of this ability, and our faith in the written word.

### 4.1 Effects on the amount of human writing

The usage of LLMs has already shown a potential to increase the efficiency of text production in the sense that text can be produced much faster and at a much lower cost than before. Style-wise, the quality is often as high as for human writing. However, the factual content is sometimes incorrect, and human control and intervention are required. As more advanced LLMs and support tools are developed, this need is likely to be reduced. Overall, the financial incentives of using LLMs are already huge, and we may soon regard “manual writing” of large texts as exotic and expensive as chopping down a forest of trees using an ax. Obviously, this will be valid in varying degrees in different areas of text production (the limitations of LLMs are discussed further on). However, it is reasonable to expect that we will see a decline in the amount of human writing, and dramatic changes in the way humans are involved in the writing process.

### 4.2 Effects on the human ability to write

There are several examples of how human skills have degraded because of technological innovations and traditional automation (Carr, 2014). The innovations of calculators, watches, GPS navigators, and auto-pilots (Garner, 2011) are examples of the former. The introduction of machines that do sewing, weaving, welding, and digging are examples of the latter - often denoted “deskilling.” When AI systems for analysis of medical X-ray images were introduced, deskilling was expected to happen to radiologists (Chockley and Emanuel, 2016). However, more recent analyses (Najjar, 2023) rather emphasize how the responsibilities of radiologists change from basic image interpretation to validation and monitoring. For skills related to writing, similar mixed effects are possible. Studies on spelling and grammar checkers in word processors suggest that the usage of such tools both motivates writers and improves writing quality (Wen and Walters, 2022). However, leaving the entire writing process to LLMs will very likely lead to a general decline in our ability to write, simply because we write less.

### 4.3 Effects on the valuation of the ability to write

The valuation and appreciation of writing skills will change, in the same way as the valuation of mental arithmetic and memorization of train times changed when we got calculators and printed timetables. A more recent example is when *googling* replaced rote learning. Who is any longer impressed by someone who knows the population figures for all of Europe's countries? And for the future, who will be impressed by someone who can formulate a detailed and lengthy appeal to the Tax Agency, or write a humorous speech for a birthday party? However, the change in valuation and appreciation can happen in several ways:

- We can regard the skill as trivial. This is how we reacted when calculators were introduced. We happily accepted that the machines do the job much better than us, but we are far from regarding them as our masters, or even intelligent. The same holds for spell-checkers with autocorrect. To some extent, this makes us feel (or maybe realize) that spelling is a trivial task. For certain tasks that LLMs can perform, we may react in the same way. Their ability to translate a text to another language, format a list of references, or summarize a research article may at first be impressive, but in not too long, we may see it as standard functionality in our word processors.
- We see the skill as not only a way to produce a text but also as a valuable process. One example is when stand-up comedians “write on stage” in interaction with the audience. The resulting text is one outcome, but so is the interactive writing process and its influence on the comedian's future work. Another example is when “the road is the goal,” for example when the task is to write a review of a research field. The writer typically learns a lot and gets new ideas while reading up on earlier work, something that is entirely lost if ChatGPT simply delivers the wanted review.
- We value achievement and talent, even if the machines are better than us. This is how we often look at athletes' performances. One example is shot put, where the task is to throw a heavy ball as far as possible. For this task, machines have been superior to humans for millennia, but this doesn't take away our admiration for good ball-throwing humans. Something similar could be possible when assessing a human's skill of writing: “Of course, an LLM would have written it much better, but coming from a *human* this book is absolutely fantastic.”
- We realize and accept that the LLMs are superior to us humans in most types of writing, which we also regard as a very valuable skill. However, we can react to this insight in different ways. Either by focusing on feeling inferior to the machines, or by enjoying having created machines that can do also intellectual work for us.

### 4.4 Effects on our faith in the written word

Our faith in the written word itself will be put to the test. Already in 2026, it is estimated that at least 90% of all content on the Internet will be created by computers (Europol, 2022). Even if we solve the problems that the LLMs produce outright inaccuracies and fabrications, much of what is written will be intentionally incorrect because it is produced to manipulate people: to buy certain products, to hold certain opinions, and to vote a certain way. If we do not develop effective ways to distinguish credible text from outright lies, we will likely lose faith in many things written on the Internet. This is related to the problem with the so-called *deepfakes* affecting images, audio, and video content, which at worst can lead to a “post-epistemic world where it is difficult or impossible to distinguish fact from fiction” (Horvitz, 2022).

## 5 Limitations of what LLMs will and can be used for

The global long-term impact of LLMs for text production obviously depends on how much and for what the LLMs will be used. We will in this section discuss several reasons why LLMs will not, or cannot, be used for certain tasks. Some practical reasons (that may be addressed in future generations of LLMs) are:

- The generated text is sometimes incorrect (sometimes referred to as “hallucinations”) (Bubeck et al., 2023).
- The generated text is sometimes ethically or legally inappropriate or unacceptable (Zhou et al., 2023).
- Data security issues (mainly the risk of revealing proprietary information) make companies forbid usage (McGlauflin, 2023).
- The knowledge stored in an LLM is limited to the date at which the model was trained (in the case of ChatGPT, September 2021).

A more fundamental limit of what an LLM can achieve is related to the so-called *embodiment hypothesis*, which states that “intelligence emerges in the interaction of an agent with an environment …” (Smith and Gasser, 2005). If this hypothesis is correct, LLMs can only be intelligent if they are connected to sensors and actuators, thereby turning into embodied robots. There are several arguments in support of the embodiment hypothesis. *Tacit knowledge* (Polanyi, 1958) is knowledge that is difficult to write down, visualize, or transfer from one person to another. Such knowledge is not accessible for an LLM, since the texts it has been trained on, almost by definition, do not contain the necessary information. Some examples of tacit knowledge are:

- Knowledge about skills that can only be learned through interaction with the world, such as biking and playing an instrument.
- Knowledge connected to perception, which requires moving around, touching objects, and collecting data (Bajcsy et al., 2018).
- Social knowledge, such as understanding and predicting other's behaviors during social interaction, must be learned by physically engaging in social interaction.

Another fundamental limitation of LLMs is that they cannot generate text from a first-person perspective. For example, an LLM can be asked to write about life in prison, but the result would be generated by mimicking people's descriptions of life in prison, rather than being based on the LLMs' own experiences of being in prison. For human authors, this is an important distinction, and the same principle should hold for LLMs. This limitation is relevant for all writing related to experiences, emotions, and self-awareness, but obviously is less relevant for fact-based writing tasks.

Finally, training a new generation of an LLM with text partly generated by the current model is problematic for several reasons. As discussed above, text generated by an LLM is sometimes erroneous, and these errors may be passed on to the new LLM such that each new generation generates a larger portion of incorrect output than the previous one. An additional problem is denoted *model collapse* (Shumailov et al., 2023), which causes the new model to forget facts since the tails of the original statistical distribution disappear.

## 6 Alternative future scenarios

We will now investigate alternative future scenarios, based on the above predictions of the amount of human writing, humans' ability to write, and our valuation of the skill to write. Is there any hope at all for the written word if all these factors degrade, and we at the same time lose our faith in what is written? An interesting comparison can be made with the development of portrait painting when the camera was invented in the middle of the nineteenth century. After first declaring “from today painting is dead,”^6^ the artists found new ways of working. Impressionists and cubists did not illustrate an objective reality but rather how an observer experiences reality. Is a similar strategy possible for preserving the value of the human written word? I asked ChatGPT “What is the literary equivalent of cubism and impressionism?” and received the following answer: “Modernist writers, such as James Joyce and Virginia Woolf, explored subjective experiences and streams of consciousness, similar to cubism's and impressionism's focus on capturing inner experiences and fragmented perspectives.” So that strategy seems to be well researched already, and ChatGPT knows what it entails. Interesting and impressive as such, but a different strategy is obviously required. Perhaps a “human” vocabulary or a structure of sentences that does not exist in the texts used to train the LLMs and therefore is not produced by them. Human written text will then be rare, recognizable, and maybe also appreciated for its origin and special form.

**Figure 2:**
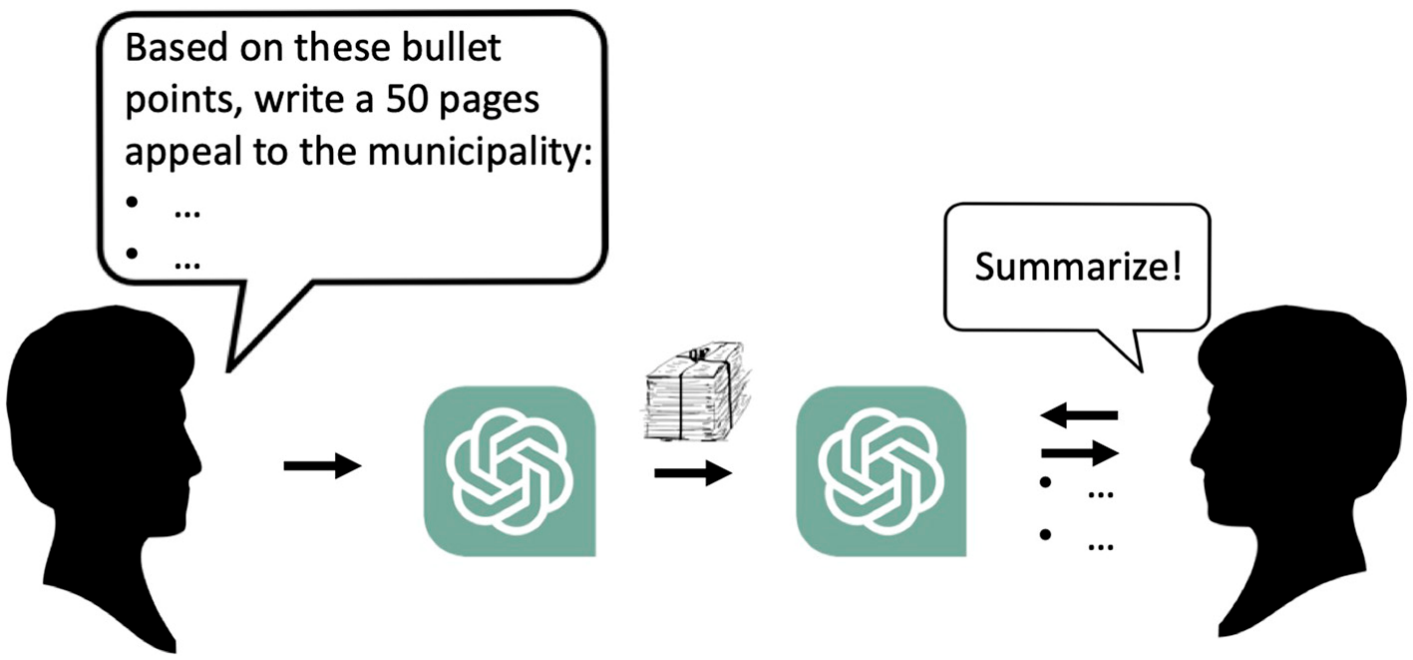
New envisioned ways of future text-based communication between people. Both writer and reader use an LLM as a tool to simplify their work.

In an apocalyptic future scenario, humans will write less and less, and gradually lose the skill to write. To the extent that we communicate in writing, systems like ChatGPT will “help,” both with writing and reading. This may create new types of human-human communication. Let us, for example, imagine how an outraged citizen asks ChatGPT to, based on a given bullet list, write a 50-page appeal to the building committee that refused to grant a building permit (Figure 2). The building committee uses ChatGPT to summarize the appeal - into a bullet list that may, or may not, correspond to the outraged citizen's original list. Besides being an odd transformation of communication between humans, it may also affect our thinking. If the citizen did not even read the 50-page-long argumentation, they are also ignorant about the content. Put differently, as a correct inference from Tegnér's “what is unclearly said is unclearly thought”: “what is not said at all is not thought at all.”

Moving even closer to the apocalypse, our knowledge and cognitive abilities may be affected by the LLMs taking over more and more of not only pure text production but also the gathering and processing of information. We previously mentioned how ChatGPT can easily be told to generate a review of a research field, and how this spares us not only work but also valuable knowledge, that we cannot even recover by thoroughly reading the generated review. Carr (2008) asked “Is Google Making Us Stupid?”, and the same question should be asked about the expected impact of LLM technology.

In a less apocalyptic scenario, AI does not take over all writing, possibly due to inherent limitations of what can be achieved. Instead, AI rather helps us humans take our writing to a higher level. Much like ancient writers called upon their muses for inspiration to start writing, we “invoke” ChatGPT for guidance on both form and content. Humans' (so far) superior creativity, common sense, and understanding of human emotions will then be combined with the AI's superior ability to identify, retrieve, and adapt previously written material.

Where we eventually end up on the apocalypse scale depends on how much writing we hand over to the LLMs. In addition, we can react to this hand-over in different ways, as discussed in Section 4.3. In any case, an open debate is essential and should range from cheering crowds embracing the many exciting possibilities, to pessimists focusing on the potential risks. Further development and societal integration of the LLMs must consider all perspectives to both minimize risks and maximize opportunities.

## Data availability statement

The original contributions presented in the study are included in the article/supplementary material, further inquiries can be directed to the corresponding author.

## Author contributions

TH: Writing—original draft, Writing—review & editing.

## Footnotes

^1^https://www.nytimes.com/2023/05/30/technology/ai-threat-warning.html#:~:text=Leaders%20from%20OpenAI%2C%20Google%20DeepMind,as%20pandemics%20and%20nuclear%20weapons/s (accessed October 19, 2023).

^2^https://renaissancerachel.com/best-ai-novel-writing-software/ (accessed October 19, 2023).

^3^https://aicontentfy.com/en/blog/future-of-creative-writing-with-ai-technology/ (accessed October 19, 2023).

^4^https://www.unitedrobots.ai/ (accessed October 19, 2023).

^5^https://www.ap.org/discover/artificial-intelligence/ (accessed October 19, 2023).

^6^https://www.nytimes.com/1972/04/23/archives/from-today-painting-is-dead.html/ (accessed October 19, 2023).
